# Correlates of muscle strengthening exercise in adolescents: A machine learning based analysis

**DOI:** 10.1371/journal.pone.0354343

**Published:** 2026-08-12

**Authors:** Yiwei Ge, Jingkun Bi

**Affiliations:** 1 Teaching and Research Unit of Physical Education, Department of Public Teaching, Shanghai Institute of Tourism, Shanghai, China; 2 Teaching and Research Unit of Physical Education, Department of General Studies, Shanghai Urban Construction Vocational College, Shanghai, China; Università degli Studi di Milano: Universita degli Studi di Milano, ITALY

## Abstract

**Background:**

Muscle-strengthening exercise (MSE) is a critical component of adolescent health, yet its correlates remain less understood than those of aerobic activity. This study aimed to identify key correlates of meeting MSE guidelines among U.S. adolescents using an explainable machine-learning approach.

**Methods:**

This cross-sectional study used data from the 2023 National Youth Risk Behavior Survey (YRBS), a nationally representative sample of U.S. high school students (n = 20,103). An eXtreme Gradient Boosting (XGBoost) classifier was developed to predict adherence to the MSE guideline (≥ 3 days/week) using sociodemographic, behavioural, dietary, and psychosocial predictors. Model performance was evaluated using the area under the curve (AUC) and accuracy. SHapley Additive exPlanations (SHAP) and partial dependence plots were employed to interpret feature importance and functional relationships.

**Results:**

Overall, 61.8% of participants in the analytic sample met the MSE guideline. The XGBoost model demonstrated robust predictive performance (AUC = 0.835; Accuracy = 0.769). Feature importance analysis identified moderate-to-vigorous physical activity (MVPA), sex, and fruit intake as the top predictors. SHAP summary plots revealed that higher MVPA, male sex, and healthier dietary behaviours were associated with a higher probability of meeting the guideline, while poorer mental health was linked to lower adherence.

**Conclusion:**

MSE participation is not an isolated behaviour but is strongly clustered with aerobic activity and a broader healthy lifestyle profile. The findings highlight significant sex disparities and the role of psychosocial well-being in strengthening behaviours. Explainable machine learning provides a useful framework for identifying and prioritising correlates associated with MSE participation, which may help inform future hypothesis-driven research and potential public health strategies.

## 1. Introduction

Over recent decades, rising levels of overweight and obesity among children and adolescents have become a major global public health concern [1,2]. Estimates from international pooled analyses indicate that youth adiposity has increased substantially in both high- and middle-income countries, reflecting widespread changes in food environments, sedentary behaviour patterns and broader socioeconomic conditions [1,3]. Excess adiposity during adolescence has important health consequences. Adolescents with overweight or obesity are at increased risk of metabolic dysfunction, insulin resistance and type 2 diabetes, and they tend to exhibit less favourable cardiometabolic risk profiles that may track into adulthood [4–6]. The burden is not limited to physical health. A growing literature highlights the close link between weight-related problems and mental health challenges, including higher risks of depressive symptoms, anxiety, emotional difficulties and lower psychosocial functioning [7–9]. Against this backdrop, identifying modifiable movement behaviours that support healthy development has become increasingly important [2,10]. Identifying correlates of health-enhancing movement behaviours may support more targeted prevention efforts [11,12].

Muscle-strengthening exercise (MSE) is a modifiable movement behaviour explicitly highlighted in global health recommendations [13]. International guidelines consistently recommend that adolescents perform MSE on at least three days per week [13,14]. MSE (e.g., push-ups, resistance training and other bodyweight exercises) is an essential but often overlooked component of youth physical activity that supports muscular strength and musculoskeletal health, alongside potential benefits for motor competence and functional capacity [6,15]. A growing body of empirical research underscores the value of MSE for adolescent physical health. Observational studies involving large school-based samples have reported inverse associations between MSE frequency and overweight or obesity [16]. Meta-analytical evidence further suggests that youth with higher levels of muscle-strengthening activity exhibit more favourable cardiometabolic profiles, lower adiposity and greater bone density [6]. These benefits extend beyond physical health, as accumulating evidence indicates that regular MSE participation is associated with better psychological outcomes, including reduced depressive symptoms [7], lower anxiety [9] and enhanced subjective wellbeing and resilience [8,17]. North American research similarly shows that adolescents who perform muscle-enhancing behaviours tend to report more favourable psychosocial adjustment [18,19].

Despite the recognised benefits outlined above, participation among adolescents remains suboptimal [20]. Studies consistently show that boys are more likely than girls to meet MSE guidelines, and that disparities exist across racial/ethnic groups, weight categories and levels of sport involvement [11,12,21]. MSE participation also tends to cluster with other lifestyle behaviours; for example, adolescents who regularly engage in MSE are more likely to be physically active, to exhibit healthier dietary patterns and to report attempts to manage body weight [22–24]. Such clustering suggests that MSE is not an isolated behaviour but part of a broader behavioural profile shaped by social, psychological and environmental factors.

However, current evidence also reveals several persistent limitations. Much of the existing literature relies on traditional regression-based approaches, which typically model only a small set of variables and assume linear associations [16,18,19]. Although these methods provide valuable insights into specific predictors, they are less suited to capturing the complex, multidimensional and potentially non-linear relationships that characterise adolescent movement behaviours [25,26]. Moreover, prior research has tended to focus on isolated correlates, such as depressive symptoms, weight status or dietary behaviours, without simultaneously incorporating a comprehensive set of demographics, behavioural and psychosocial variables [12,27]. As a result, little is known about the relative importance of these factors in explaining adherence to MSE recommendations.

Epistemologically, adolescent health and movement behaviours do not occur in a vacuum; rather, they exist within a complex, interacting socio-ecological system. Traditional regression models, which typically assume independent and linear additive effects, may oversimplify the reality where lifestyle factors, such as dietary patterns, psychological well-being, and other forms of physical activity, strongly cluster and interact synergistically. Machine learning (ML) offers a promising way to address these gaps by enabling high-dimensional modelling, flexible detection of non-linear patterns and interpretable ranking of predictor importance [25,26]. Yet applications of ML to adolescent MSE remain rare. Although the U.S. Youth Risk Behavior Survey (YRBS) provides a nationally representative dataset with rich behavioural, psychosocial and demographic indicators, existing YRBS-based studies have examined only a narrow subset of variables and have relied exclusively on conventional statistical approaches [18,19,24]. Consequently, a comprehensive, data-driven understanding of the multilevel correlates of adolescent MSE in the United States is still lacking.

The aim of this study was to use nationally representative data from the YRBS to examine the correlates of meeting the MSE guideline among U.S. adolescents using an explainable machine-learning framework. Specifically, this study addressed the following research question: which demographic, behavioural, dietary, and psychosocial factors are most strongly associated with meeting the recommended level of muscle-strengthening exercise among adolescents? By using eXtreme Gradient Boosting (XGBoost) and SHapley Additive exPlanations (SHAP) analyses, the study aimed to clarify which factors are most influential and to provide evidence that may guide more targeted strategies to promote MSE among adolescents.

## 2. Materials and methods

### 2.1 Study design and data source

This cross-sectional study used data from the 2023 Youth Risk Behavior Survey (YRBS), a nationally representative school-based surveillance system conducted by the U.S. Centers for Disease Control and Prevention (CDC) [3]. The YRBS employs a three-stage cluster sampling design to generate representative estimates for students in grades 9–12 in the United States. The present study used the publicly available, de-identified 2023 national YRBS dataset. The original YRBS protocol was approved by the institutional review boards of the CDC and ICF, the survey contractor. Participation was anonymous and voluntary, and local parental permission procedures were followed before survey administration. Because the present study involved only secondary analysis of preexisting, publicly available, and completely de-identified data, it was granted an exemption from ethical review by the Academic Committee of the Shanghai Institute of Tourism (No. SIT-AC-2025–002), and no additional informed consent was required.

No participants were excluded a priori. To maximize statistical power and avoid potential bias introduced by listwise deletion, all 20,103 high school students who participated in the 2023 YRBS were retained in the analytic sample. Missing data were handled using imputation techniques described in Section 2.3. It should also be noted that the complex survey weights provided by the YRBS were not applied in this analysis. Machine learning algorithms such as XGBoost are designed primarily for individual-level prediction and pattern recognition rather than for population parameter estimation. Therefore, the model was trained and evaluated using unweighted observations. This decision allows the algorithm to focus on identifying predictive patterns across individuals rather than producing weighted national prevalence estimates. The dataset was accessed for research purposes on February 5, 2025. The authors had no access to any information that could identify individual participants during or after the data collection process.

### 2.2 Variables

#### 2.2.1 Outcome variable.

The primary outcome was adherence to the MSE guideline. Students reported the frequency of activities designed to build muscular strength (e.g., push-ups, sit-ups, weight training) over the previous 7 days. Consistent with international guidelines, responses were binary-coded as meeting the recommendation (≥ 3 days/week) or not meeting the recommendation (0–2 days/week) [2].

#### 2.2.2 Predictor variables.

Candidate predictors were defined a priori to reflect key domains of adolescent characteristics and context (see S1 Table for the full list of variables). Generally, variables were grouped into four domains: (1) sociodemographic factors (age, sex, and race/ethnicity); (2) weight status, derived from BMI-for-age percentiles; (3) lifestyle and health behaviours (e.g., dietary intake, physical activity, sleep duration, screen time); and (4) psychosocial and social-context indicators (e.g., depressive symptoms, bullying victimisation, school belonging).

Race/ethnicity (source variable: raceeth) was harmonised into four categories: White; Black or African American; Hispanic/Latino (including multiple Hispanic/Latino); and All Other Races (American Indian/Alaskan Native, Asian, Native Hawaiian/Other Pacific Islander, and multiple non-Hispanic/Latino). Weight status was derived from BMI-for-age percentiles (BMIPCT) using CDC cut-points and categorised as underweight (<5th percentile), healthy weight (5th to <85th), overweight (85th to <95th), and obesity (≥95th) [28]. Predictors were otherwise modelled using the survey’s original categorical or ordinal response options, with limited recoding applied to align response categories and reduce sparse cells where necessary.

In this study, dietary indicators (e.g., fruit and salad intake) were included as candidate predictors within the machine-learning model to capture behavioural patterns associated with muscle-strengthening exercise. In predictive modelling, variables are selected based on their ability to improve classification performance rather than on an assumed causal direction [25]. Previous research shows that dietary behaviours, physical activity, and weight-control practices commonly cluster among adolescents and often reflect a broader health-oriented lifestyle [23,24]. Therefore, dietary indicators were incorporated to represent this behavioural context. However, because the data are cross-sectional, the temporal ordering between diet and muscle-strengthening exercise cannot be determined. Reverse or bidirectional relationships remain possible.

### 2.3 Statistical analysis

All analyses followed a structured workflow consisting of data preprocessing, model development, model evaluation, and explainability analysis.

#### 2.3.1 Data preprocessing.

Prior to model training, all nominal categorical variables were converted into dummy variables using one-hot encoding, while ordinal variables were encoded as integer values to preserve their natural ranking structure. To prevent information leakage, the raw dataset was first randomly divided into a training set (70%) and an independent testing set (30%) before any imputation procedures were performed. Missing data were handled using multiple imputation by chained equations (MICE) [29]. The imputation procedure was conducted independently within the training and testing datasets. This approach ensured that information from the testing set did not influence the model development process. MICE was selected because it performs well with datasets containing mixed variable types (categorical and ordinal), which are common in the YRBS survey. Alternative approaches such as KNNImpute and MissForest were considered; however, MissForest can be computationally intensive for large datasets, while KNNImpute may be less suitable for complex categorical structures. To support consistent model interpretation, we used the 20th imputed dataset from the MICE procedure, after confirming stable Markov chain convergence through trace plots [30]. Currently, no standardized approach exists for combining SHAP explainability outputs across multiple imputed machine learning models. Therefore, using a single converged imputation dataset provides a practical solution for generating interpretable SHAP results. All candidate predictors defined a priori were included in the model without additional feature selection. The XGBoost algorithm applies internal regularization, which helps control model complexity and automatically adjusts the contribution of correlated predictors.

#### 2.3.2 Model development.

An eXtreme Gradient Boosting (XGBoost) classifier was used to estimate the probability of meeting the MSE recommendation (≥3 days per week) [31]. Hyperparameters, including the learning rate (eta), maximum tree depth, and subsampling ratios, were optimized using a step-wise, granular grid search approach combined with 5-fold cross-validation within the training dataset. This iterative process involved an initial coarse-grained search over a broad parameter space to identify the optimal region, followed by a second stage of fine-grained refinement around the identified optima. This granular strategy was employed to ensure that the global optimum between initial grid lines was not missed, thereby improving model stability and reducing the risk of overfitting. The optimal hyperparameters were selected based on the highest mean validation area under the receiver operating characteristic curve (AUC). AUC was chosen because it measures classification performance across all possible probability thresholds and is less sensitive to class imbalance than simple accuracy. To reduce the risk of overfitting, early stopping was applied during model training. The boosting process was terminated when the validation AUC failed to improve for 20 consecutive iterations. This procedure, together with tree-depth constraints and subsampling, improves the generalizability of the final model.

#### 2.3.3 Model evaluation.

Predictive performance was evaluated using the independent testing set. Performance metrics included accuracy, sensitivity, specificity, and AUC, with 95% confidence intervals (CIs) estimated using bootstrap resampling (1,000 iterations).

#### 2.3.4 Model explainability.

To interpret the trained model, we applied SHapley Additive exPlanations (SHAP), a model-agnostic method derived from cooperative game theory [32]. SHAP quantifies how much each predictor contributes to the predicted probability for a given observation. In simple terms, SHAP values represent the change in the model output associated with a specific feature compared with the average prediction. In this study, mean absolute SHAP values (Mean |SHAP|) were used to quantify the overall importance of each predictor across all observations. Larger values indicate stronger influence on model predictions. We also generated SHAP summary plots, which display both the magnitude and direction of each variable’s contribution across the dataset. In these plots, each point represents an individual observation, and the color scale indicates the relative level of the predictor variable. To further examine non-linear relationships between predictors and the probability of meeting the MSE guideline, partial dependence plots (PDPs) were produced. PDPs show the average predicted probability of the outcome across the range of a predictor while holding other variables constant. Data management was conducted using SPSS version 27 and Stata version 17, and all machine learning analyses were performed in R version 4.4.0.

## 3. Results

As shown in Table 1, 61.8% of respondents in the analytic sample reported meeting the MSE guideline of at least three days per week. The proportion of participants meeting the guideline decreased slightly with age and was substantially higher among boys (72.2%) than girls (51.0%). Adolescents identifying as White showed the highest adherence, whereas Black or African American youth had the lowest. These descriptive differences may reflect broader social and contextual factors, which are considered in the Discussion.

**Table 1:** Descriptive statistics of meeting MSE guidelines by age, sex, and race.

Variable	Not meeting	Meeting	Total
**Total**	7,684 (38.2%)	12,419 (61.8%)	20,103 (100%)
			
**Age**			
12 years old	13 (29.5%)	31 (70.5%)	44 (0.2%)
13 years old	11 (33.3%)	22 (66.7%)	33 (0.2%)
14 years old	962 (37.5%)	1,607 (62.5%)	2,569 (12.8%)
15 years old	2,003 (35.6%)	3,621 (64.4%)	5,624 (28.0%)
16 years old	2,010 (38.6%)	3,198 (61.4%)	5,208 (25.9%)
17 years old	1,824 (40.9%)	2,634 (59.1%)	4,458 (22.2%)
18 years old or older	861 (39.7%)	1,306 (60.3%)	2,167 (10.8%)
**Sex**			
Girls	4,841 (49.0%)	5,043 (51.0%)	9,884 (49.2%)
Boys	2,843 (27.8%)	7,376 (72.2%)	10,219 (50.8%)
**Race**			
White	3,697 (36.7%)	6,373 (63.3%)	10,070 (50.1%)
Black or African American	781 (43.6%)	1,010 (56.4%)	1,791 (8.9%)
Hispanic/Latino	1,581 (39.6%)	2,413 (60.4%)	3,994 (19.9%)
All Other Races	1,625 (38.3%)	2,623 (61.8%)	4,248 (21.1%)

The machine-learning model demonstrated satisfactory predictive performance (Table 2), with an accuracy of 0.769 (95% CI: 0.758–0.779), F1 score = 0.808, and area under the curve (AUC) = 0.835 (95% CI: 0.825–0.846). A baseline multivariate logistic regression model achieved comparable predictive performance (Accuracy = 0.781; AUC = 0.833). However, because our primary research questions focused on uncovering non-linear threshold effects and multidimensional interactions without a priori specification, the XGBoost-SHAP framework was retained as the primary analytic engine. Furthermore, the confusion matrix for the XGBoost model on the independent testing set revealed 2,927 true positives, 1,709 true negatives, 598 false positives, and 797 false negatives. These metrics indicate that the model achieved robust discrimination and balanced sensitivity and specificity.

**Table 2:** Performance metrics of the machine learning model.

Metric	Value
Accuracy (95% CI)	0.769 (0.758–0.779)
Kappa	0.518
Sensitivity (Recall)	0.786 (0.772–0.799)
Specificity	0.741 (0.722–0.759)
Positive Predictive Value (Precision)	0.830
Negative Predictive Value	0.682
Balanced Accuracy	0.763
F1 Score	0.808
AUC	0.835 (0.825–0.846)

**Fig 1:**
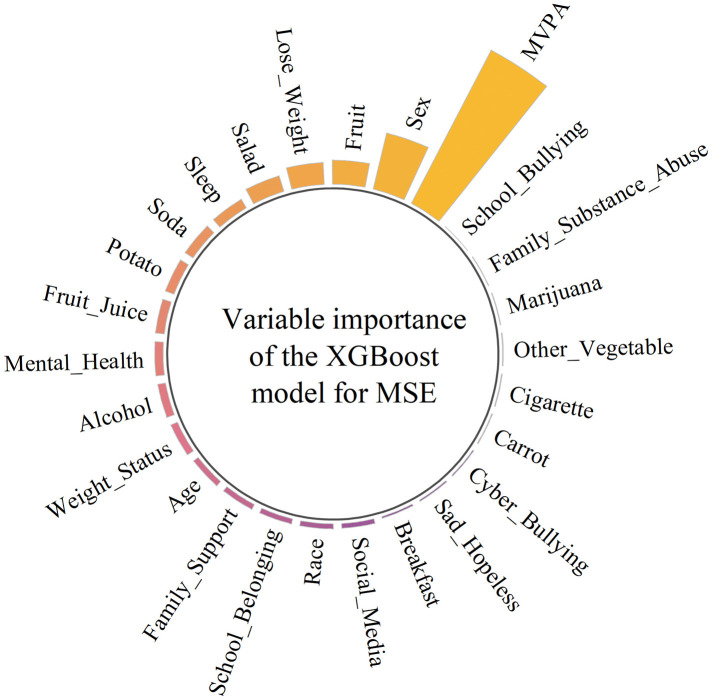
Variable importance of the XGBoost model for predicting muscle-strengthening exercise. (Note: The bar chart ranks the predictors based on their relative importance score in the final model).

Feature importance analysis revealed that moderate-to-vigorous physical activity (MVPA), sex, and fruit intake were the most influential predictors of meeting MSE guidelines (Fig 1). To quantify absolute importance, we extracted the mean absolute SHAP values (Mean |SHAP|), which represent the average magnitude of a feature’s impact on the model’s raw output margin. MVPA was the dominant predictor (Mean |SHAP| = 1.058), followed by sex (0.353), fruit intake (0.148), and attempts to lose weight (0.134). Additional contributors included salad consumption, sleep, soda, potato, fruit juice and mental health status, suggesting that lifestyle, nutritional, and psychological factors jointly shaped MSE participation.

**Fig 2:**
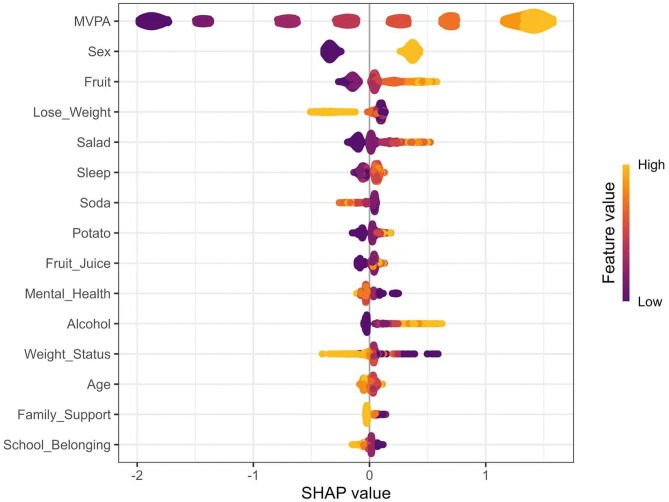
SHAP summary plot of predictors for muscle-strengthening exercise. (Note: This plot illustrates the impact of top features on model output, where yellow indicates high feature values and purple indicates low values).

To better interpret the direction and magnitude of each feature’s effect, a SHAP summary plot was generated (Fig 2). Higher MVPA levels and male sex were associated with increased probabilities of meeting the MSE guideline, while poorer mental health and unhealthy dietary behaviours were linked to lower adherence. The wide spread of SHAP values for MVPA and sex indicates their dominant influence in the model.

**Fig 3:**
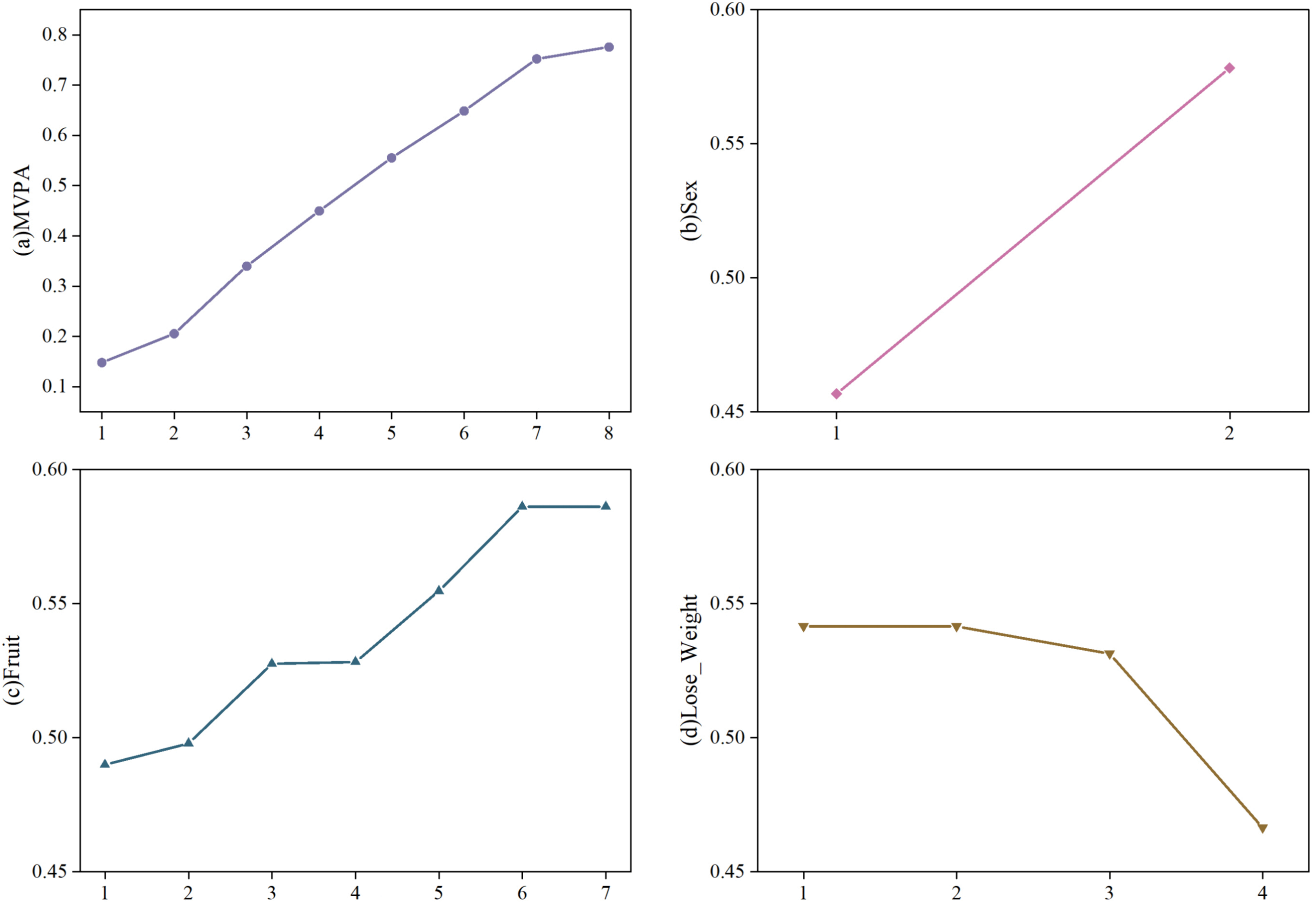
Partial dependence plots of top predictors. (Note: These plots display the marginal relationship between the top predictors and the model-predicted probability of meeting muscle-strengthening exercise guidelines).

Partial dependence plots (Fig 3) further visualised these relationships, showing that the likelihood of meeting MSE guidelines increased steadily with higher MVPA and fruit consumption. Boys consistently demonstrated higher predicted probabilities than girls, and adolescents attempting to lose weight displayed a modestly elevated likelihood compared to those not trying.

## 4. Discussion

### 4.1 Principal findings

Explainable ML analyses indicated that MVPA, sex, and fruit intake were the most influential correlates of meeting the MSE guideline. Other variables also contributed, including attempts to lose weight, salad intake, and mental health indicators. Across explainability outputs, higher MVPA and being male were associated with a higher probability of meeting the guideline, whereas poorer mental health was associated with a lower probability. Dietary indicators showed a positive pattern, which may reflect clustering with a broader healthy lifestyle profile. In addition, 61.8% of participants in the analytic sample met the MSE recommendation (≥3 days/week). The XGBoost classifier showed good discrimination (accuracy 0.769, AUC 0.835). Although a baseline logistic regression yielded comparable predictive accuracy, the XGBoost–SHAP framework remains valuable for exploratory analysis. Rather than identifying causal determinants, its primary contribution lies in identifying potential correlates and revealing non-linear functional patterns (e.g., the dose–response curves observed in the partial dependence plots) without the need to pre-specify interaction terms in traditional regression models. This approach therefore provides a data-driven framework for prioritising candidate correlates that may warrant further investigation in longitudinal research. Furthermore, while the high predictive metrics might suggest a potential risk of overfitting, the robustness and generalizability of our model are supported by the smooth and logical functional transitions observed in the SHAP and partial dependence plots (Figs 2 and 3). In our analysis, the predicted probability of meeting MSE guidelines exhibits a clear, monotonic relationship with key correlates such as MVPA and dietary intake. The absence of erratic fluctuations, jittery spikes, or illogical zig-zags in these interpretability curves demonstrates that the XGBoost model successfully captured the underlying “true signal” of adolescent health behaviors rather than memorizing random noise or outliers in the training set. This stability, combined with the rigorous application of the early stopping technique and step-wise granular hyperparameter tuning, confirms that the identified behavioral profiles represent meaningful associations rather than a result of over-fitting.

### 4.2 Interpretation of findings

#### 4.2.1 MVPA.

MVPA emerged as the strongest correlate of meeting the MSE guideline in our explainable ML models. This pattern is consistent with youth movement guidelines that position aerobic activity and muscle-strengthening as complementary targets rather than independent behaviours [2,14]. Evidence from U.S. surveillance data also shows substantial overlap between aerobic and muscle-strengthening behaviours among adolescents [19].

One plausible interpretation is behavioural clustering. Adolescents who accumulate higher levels of MVPA may also participate in activity settings where resistance-type exercises are available or encouraged [2]. Previous YRBS-based analyses show that muscle-strengthening exercise frequently co-occurs with vigorous activity within broader activity profiles [24]. Large school-based studies also report that associations between MSE and weight status differ depending on whether adolescents meet general physical activity guidelines, suggesting that these behaviours often co-exist within an overall activity pattern [16]. Our partial dependence results similarly indicate that higher MVPA levels are associated with a higher predicted probability of meeting the MSE guideline. Taken together, MVPA in this model likely reflects a broader active lifestyle context rather than an independent behavioural driver of MSE participation [19].

#### 4.2.2 Sex.

Sex was a dominant correlate in our models, with boys showing a consistently higher probability of meeting the MSE guideline. This pattern aligns with prior evidence that male adolescents report more frequent MSE and related muscle-oriented behaviours than females [18,19]. Analyses using U.S. YRBS data also indicate that boys are more likely than girls to meet MSE recommendations and to show joint adherence with aerobic activity [19].

Several mechanisms may explain this gap. Strength and muscularity are often framed as masculine ideals, which can increase encouragement and social acceptance for boys to engage in resistance-type activity [18]. These norms may be reinforced in sport and school settings where strength training is more visible or more strongly promoted for male participants [18,19]. In contrast, girls may face fewer invitations, fewer role models, and less perceived belonging in strength-focused spaces, which can reduce uptake even when opportunities exist [18]. Psychological barriers may also differ by sex. Boys may enter adolescence with greater familiarity and confidence in strength-oriented activities, while girls may be more likely to anticipate negative evaluation, feel discomfort in mixed-gender environments, or worry about appearance-related consequences of training [18]. Taken together, the sex gradient in MSE participation is more plausibly linked to modifiable social and contextual factors than to biological constraints [18].

#### 4.2.3 Dietary indicators (fruit and salad intake).

Dietary indicators, particularly fruit and salad intake, were prominent predictors in the model. Higher intake was associated with a higher predicted probability of meeting the MSE guideline. This pattern may reflect health behaviour clustering, rather than a direct dietary effect. Adolescents who report exercise-based weight control often report higher fruit and vegetable intake, consistent with a broader health-oriented behavioural profile [24]. However, this behavioral clustering may also be influenced by unmeasured or residual confounding, such as household income or neighborhood environments, which jointly shape both diet and physical activity.

A nutritional pathway is also possible but should be interpreted cautiously. Diet and physical activity are jointly related to muscle-related outcomes in youth [23], and experimental evidence suggests that fruit-derived compounds may help reduce exercise-induced muscle damage and inflammation [33]. Taken together, higher fruit and salad intake may act as a marker of better overall diet quality that supports sustained engagement in strengthening activity [23,33]. However, given the cross-sectional nature of the present data, these dietary variables are better interpreted as markers of a healthier lifestyle pattern that co-occurs with strengthening activity rather than as causal determinants of MSE participation.

#### 4.2.4 Attempts to lose weight.

Attempts to lose weight emerged as a key predictor of meeting the MSE guideline, indicating that weight-control motivation is associated with adolescents’ strengthening behaviour. Previous YRBS analyses have similarly reported that adolescents trying to lose weight are more likely to report muscle-strengthening activity [24].

Several interpretations are possible. First, MSE can be perceived as a practical strategy for changing body shape (e.g., “toning”) and increasing muscle definition, which may appeal to adolescents who are actively trying to manage weight [24]. Second, weight-related goals often co-occur with broader body-change motives, including efforts to increase muscle size or tone, and such motives are common in both boys and girls [18]. Third, there is a plausible physiological pathway: resistance training can improve muscular strength and lean mass and may support favourable body-composition and cardiometabolic profiles among youth with overweight or obesity [15,34]. This association may also reflect reverse causality and shared confounding. Adolescents with higher adiposity or muscularity-motivated may be more likely to both attempt weight loss and engage in muscle-focused training, and weight status has been linked with higher use of muscle-enhancing behaviours [18]. However, this association may also reflect reverse causation or shared underlying factors, such as body image concerns or higher adiposity, which may increase both weight-loss attempts and engagement in strengthening activity [18]. Therefore, the observed relationship should be interpreted as an association rather than evidence of a directional behavioural pathway.

#### 4.2.5 Mental health indicators.

In our model, indicators of psychological distress (e.g., low mood and suicidality) contributed meaningfully to predicting MSE guideline adherence. This aligns with evidence that adolescents who engage in MSE, especially when combined with aerobic activity, tend to report better mental health profiles.

The direction is plausibly bidirectional. On one hand, poorer mental health may reduce motivation, self-efficacy, and social participation, which can lower engagement in structured exercise contexts where MSE occurs. On the other hand, higher MSE participation is consistently associated with fewer depressive symptoms and better positive mental health outcomes in adolescent studies, suggesting potential mental-health benefits of MSE [8,17]. Taken together, the model output likely reflects that MSE sits within a broader “functioning and coping” profile, where psychological distress co-occurs with lower activity participation, while better mental health co-occurs with higher participation [7]. Given the cross-sectional design, the present findings should be interpreted as reflecting co-occurring behavioural and psychosocial patterns, rather than evidence that MSE directly improves mental health.

#### 4.2.6 Other contributing correlates.

Beyond MVPA, sex, weight-control attempts, dietary indicators, and mental health, the ML outputs suggested additional, smaller contributors: age, sleep, breakfast, sugar-sweetened beverages (soda/fruit juice), alcohol use, potato intake, family support, and school belonging.

Age showed a modest contribution, consistent with evidence that MSE participation tends to decline across adolescence and that older youth can be an at-risk group for low engagement [10]. Sleep and daily routines (sleep, breakfast) likely reflect broader lifestyle patterning rather than isolated drivers. In a large adolescent survey, longer sleep duration was associated with a higher likelihood of muscle-strengthening physical activity and a lower likelihood of breakfast skipping, alongside healthier dietary behaviours [22].

Unhealthy dietary markers (soda/fruit juice, potato) and alcohol use may capture clustering of behaviours that co-occur with lower (or less sustained) structured exercise. For example, sleep duration in adolescents has been linked with multiple health-related behaviours, including alcohol use, suggesting these behaviours form interrelated profiles rather than independent exposures [22]. Family support and school belonging likely represent access, norms, and encouragement that make MSE more feasible. Reviews highlight parental support as a key predictor of youth compliance with muscle-strengthening activities through encouragement, logistical support, and co-participation [12]. Schools are also a central setting for MSE opportunities via PE curriculum, staff, and infrastructure, and gendered access to spaces may matter [11].

Race and ethnicity also contributed modestly to the prediction of meeting the MSE guideline. Descriptive results indicated differences across racial and ethnic groups. In population health research, race and ethnicity are typically interpreted as social indicators rather than biological determinants. Differences in physical activity behaviors may reflect broader contextual conditions, including socioeconomic resources, access to sports facilities, school physical education opportunities, and neighborhood environments [10,12]. These contextual factors may shape adolescents’ opportunities and support for participating in muscle-strengthening activities. Therefore, the observed differences in this study are more likely to reflect social and environmental contexts than inherent individual characteristics.

### 4.3 Public health implications

The pattern of correlates indicates that adolescents’ MSE participation aligns with broader behavioural and psychosocial profiles rather than a single isolated factor. The strong overlap with MVPA suggests that strengthening activity often occurs within established activity contexts and routines, consistent with guidelines positioning aerobic and muscle-strengthening activity as complementary components of adolescent movement behaviour [2]. The marked sex gap highlights that participation is shaped by social norms and access, and that girls may experience distinct barriers to engaging in strength-oriented activity [18,19]. The contributions of dietary indicators, weight-control attempts, and mental health measures further suggest that MSE adherence sits within a wider “health orientation” profile, where lifestyle practices and psychological functioning co-occur [8,24]. These findings may help identify groups and behavioural contexts that warrant attention in future research. However, because the present analysis is cross-sectional, the results should be interpreted as associations rather than causal relationships, and longitudinal studies are needed to clarify temporal ordering and potential behavioural pathways.

### 4.4 Strengths and limitations

#### 4.4.1 Strengths.

This study has several strengths. It used a large sample of U.S. high-school students, providing broad coverage of U.S. high school students within the YRBS sampling frame. The availability of demographic, behavioural, dietary, and psychosocial variables enabled a broad assessment of correlates of meeting the MSE guideline. The machine-learning approach, paired with explainability tools (SHAP and partial dependence plots), supported modelling of non-linear patterns and interactions and provided interpretable summaries of the predictors most relevant to classification. Together, these features strengthen confidence in the robustness of the observed correlate patterns.

#### 4.4.2 Limitations.

Several limitations should be acknowledged. First, YRBS measures are self-reported, which may introduce recall and social desirability bias, particularly for physical activity, dietary behaviours, and MSE. Second, a significant proportion of participants had missing data for the outcome variable. Although we employed multiple imputation to mitigate bias, our reliance on a single imputed dataset to facilitate SHAP value calculation means that the variance between imputations was not accounted for, which may underestimate the standard errors. Third, the cross-sectional design precludes causal inference and limits interpretation of temporal ordering. Fourth, complex survey weights were not incorporated into the machine learning pipeline; thus, while the findings robustly rank correlates at the individual level, they may lack absolute representativeness for estimating exact national population parameters. Finally, while machine learning excels at capturing non-linear patterns, it remains an observational technique. SHAP values represent predictive contributions rather than causal effects, and the risk of residual confounding cannot be eliminated. Therefore, ML-derived importance and explainability outputs describe how predictors contribute to classification within this dataset; contributions may be shared across correlated variables, and external validation is needed to assess transportability.

## 5. Conclusion

This study identified key correlates of meeting the adolescent MSE guideline among participants in the 2023 national YRBS using an explainable machine-learning approach. MSE adherence aligned most strongly with MVPA and sex, and it also co-occurred with dietary and psychosocial indicators, suggesting that strengthening behaviour is embedded within broader lifestyle and wellbeing profiles. The explainability outputs helped prioritise correlates and describe their functional patterns associated with MSE participation. While these observational findings cannot establish causality, they provide a valuable framework to help inform future hypothesis-driven research. Future studies using longitudinal designs and more detailed MSE measures are needed to clarify temporal ordering and to support targeted strategies for groups with lower participation.

## Supporting information

S1 Table Description of variables used in the analysis. (DOCX)

S1 File R code used for data processing and machine learning analyses. (R)

S2 File Underlying data used in the analysis. (XLSX)
