# Supplementary material for: Correlates of muscle strengthening exercise in adolescents: A machine learning based analysis
Source: PLoS One. 2026 Aug 12;21(8):e0354343. doi: 10.1371/journal.pone.0354343 (PMC13465836; doi:10.1371/journal.pone.0354343)
Supplement: Supplementary file 1 [file pone.0354343.s001.docx]

**Supplementary Table S1. Description of variables used in the analysis.**

| **Variable Name** | **YRBS Source** | **Question / Definition** | **Response Categories / Coding** |
| --- | --- | --- | --- |
| ***Outcome*** |  |  |  |
| MSE | Q97 | During the past 7 days, on how many days did you do exercises to strengthen or tone your muscles, such as push-ups, sit-ups, or weight lifting? | Binary:  0 = Not meeting guidelines (0–2 days)  1 = Meeting guidelines (≥3 days) |
| ***Sociodemographic*** |  |  |  |
| Age | Q1 | How old are you? | 12 years old or younger to 18 years old or older |
| Sex | Q2 | What is your sex? | Female; Male |
| Race | Raceeth | Derived from Q4 (Hispanic ethnicity) and Q5 (Race). | Harmonised into 4 categories:  1. White  2. Black or African American  3. Hispanic/Latino  4. All Other Races (Am. Indian/Alaska Native, Asian, Native Hawaiian/PI, Multiple races) |
| ***Weight Status*** |  |  |  |
| Weight Status | bmipct | Derived from BMI percentile by Q1 (age) and Q2 (sex). | Categorised as:  1. Underweight (<5th)  2. Healthy weight (5th to <85th)  3. Overweight (85th to <95th)  4. Obesity (≥95th) |
| ***Lifestyle & Health*** |  |  |  |
| Fruit Juice | Q68 | During the past 7 days, how many times did you drink 100% fruit juices such as orange juice, apple juice, or grape juice? (Do not count punch, Kool-Aid, sports drinks, or other fruit-flavored drinks.) | Ordinal: Did not drink to 4 or more times per day |
| Fruit | Q69 | During the past 7 days, how many times did you eat fruit? | Ordinal: Did not eat fruit to 4 or more times per day |
| Salad | Q70 | During the past 7 days, how many times did you eat green salad? | Ordinal: Did not eat salad to 4 or more times per day |
| Potato | Q71 | During the past 7 days, how many times did you eat potatoes? | Ordinal: Did not eat potatoes to 4 or more times per day |
| Carrot | Q72 | During the past 7 days, how many times did you eat carrots? | Ordinal: Did not eat carrots to 4 or more times per day |
| Other Vegetable | Q73 | During the past 7 days, how many times did you eat other vegetables? | Ordinal: Did not eat other vegetables to 4 or more times per day |
| Soda | Q74 | During the past 7 days, how many times did you drink a can, bottle, or glass of soda or pop, such as Coke, Pepsi, or Sprite? (Do not count diet soda or diet pop.) | Ordinal: Did not drink soda to 4 or more times per day |
| Breakfast | Q75 | During the past 7 days, on how many days did you eat breakfast? | Ordinal: 0 days to 7 days |
| MVPA | Q76 | During the past 7 days, on how many days were you physically active for a total of at least 60 minutes per day? | Ordinal: 0 days to 7 days |
| Sleep | Q85 | On an average school night, how many hours of sleep do you get? | Ordinal: 4 or less hours to 10 or more hours |
| Social Media | Q80 | How often do you use social media? | Ordinal: Do not use to More than once an hour |
| Cigarette | Q33 | During the past 30 days, on how many days did you smoke cigarettes? | Ordinal: 0 days to All 30 days |
| Alcohol | Q42 | During the past 30 days, on how many days did you have at least one drink of alcohol? | Ordinal: 0 days to All 30 days |
| Marijuana | Q48 | During the past 30 days, how many times did you use marijuana? | Ordinal: 0 times to 40 or more times |
| Lose Weight | Q67 | Which of the following are you trying to do about your weight? | Categorical: Lose weight, Gain weight, Stay same, Not trying |
| ***Psychosocial*** |  |  |  |
| School Bullying | Q24 | During the past 12 months, have you ever been bullied on school property? | Binary: Yes / No |
| Cyber Bullying | Q25 | During the past 12 months, have you ever been electronically bullied? | Binary: Yes / No |
| Sad Hopeless | Q26 | During the past 12 months, did you ever feel so sad or hopeless almost every day for two weeks or more in a row that you stopped doing some usual activities? | Binary: Yes / No |
| Mental Health | Q84 | During the past 30 days, how often was your mental health not good? | Ordinal: Never to Always |
| School Belonging | Q103 | Do you agree or disagree that you feel close to people at your school? | Ordinal: Strongly agree to Strongly disagree |
| Family Support | Q99 | During your life, how often has there been an adult in your household who tried hard to make sure your basic needs were met, such as looking after your safety and making sure you had clean clothes and enough to eat? | Ordinal: Never to Always |
| Family Substance | Q100 | Have you ever lived with a parent or guardian who was having a problem with alcohol or drug use? | Binary: Yes / No |
